# State-Level Economic Costs of Opioid Use Disorder and Fatal Opioid Overdose — United States, 2017

**DOI:** 10.15585/mmwr.mm7015a1

**Published:** 2021-04-16

**Authors:** Feijun Luo, Mengyao Li, Curtis Florence

**Affiliations:** 1 Division of Injury Prevention, National Center for Injury Prevention and Control, CDC.

Approximately 47,000 persons in the United States died from an opioid-involved overdose in 2018 (*1*), and 2.0 million persons met the diagnostic criteria for an opioid use disorder in 2017 (*2*). The economic cost of the U.S. opioid epidemic in 2017 was estimated at $1,021 billion, including cost of opioid use disorder estimated at $471 billion and cost of fatal opioid overdose estimated at $550 billion (*3*). CDC used national-level cost estimates to estimate the state-level economic cost of opioid use disorder and fatal opioid overdose during 2017. Cases and costs of state-level opioid use disorder and fatal opioid overdose and per capita costs were calculated for each of the 38 states and the District of Columbia (DC) that met drug specificity requirements for mortality data (*4*). Combined costs of opioid use disorder and fatal opioid overdose (combined costs) varied substantially, ranging from $985 million in Wyoming to $72,583 million in Ohio. Per capita combined costs also varied considerably, ranging from $1,204 in Hawaii to $7,247 in West Virginia. States with high per capita combined costs were mainly in two regions: the Ohio Valley and New England. Federal and state public health agencies can use these data to help guide decisions regarding research, prevention and response activities, and resource allocation.

Estimated case counts of state-level opioid use disorder were extracted from the National Survey on Drug Use and Health (NSDUH) 2-Year Restricted-Use Data Analysis System (2016–2017) provided by the Substance Abuse and Mental Health Services Administration (*5*). NSDUH is a nationally representative sample of the U.S. civilian noninstitutionalized population aged ≥12 years. Cases of opioid use disorder were identified by using questions on opioid abuse or dependence during the past year.* Case counts of state-level fatal opioid overdose and population estimates in 2017 were extracted from CDC’s WONDER database (*6*). Cases of fatal opioid overdose were identified using *International Classification of Diseases, Tenth Revision* underlying cause-of-death codes X40–X44, X60–X64, X85, and Y10–Y14 and then multiple causes-of-death codes T40.0–T40.4 and T40.6.^†^ This report is limited to DC and the 38 states that met the requirement that at least one specific drug is named on the death certificate (*4*).

Cost per case of opioid use disorder ($221,219) was derived by dividing the total U.S. cost of opioid use disorder ($470,975 million) during 2017 by the number of opioid use disorder cases the same year (2.129 million) (*3*). Cost per case of fatal opioid overdose ($11.548 million) was derived by dividing the total cost of fatal opioid overdose ($549,691 million) by the number of fatal opioid overdose cases (47,600) (*3*). State-level cost of opioid use disorder was calculated by multiplying the U.S. cost of opioid use disorder per case by the number of cases of opioid use disorder in each state. State-level cost of fatal opioid overdose was calculated by multiplying the U.S. cost of fatal opioid overdose per death by the number of deaths in each state. To facilitate comparison across states, CDC divided state-level combined costs of opioid use disorder and fatal opioid overdose by state population to generate per capita costs. The 38 states and DC were ranked by per capita combined costs. Cost components of opioid use disorder and fatal opioid overdose include the costs of health care, substance use treatment, criminal justice, lost productivity, reduced quality of life, and the value of statistical life lost. These components were calculated by multiplying the number of state cases of opioid use disorder or fatal opioid overdose by national cost estimates per case for each component (*3*).^§^

Cases of opioid use disorder and fatal opioid overdose varied substantially among states, and the combined costs ranged from $985 million in Wyoming to $72,583 million in Ohio (Table 1). Per capita combined costs also varied widely among states, ranging from $1,204 in Hawaii to $7,247 in West Virginia. The state-level per capita combined costs exhibited geographic patterns (Figure); states with high per capita combined costs were located mainly in the Ohio Valley and New England. Three adjacent states in the Ohio Valley (West Virginia, Ohio, and Kentucky) had the first, second, and fourth highest per capita combined costs ($7,247, $6,226, and $5,491, respectively). Per capita costs of fatal opioid overdose were highest in West Virginia ($5,298) and Ohio ($4,252). Per capita combined costs in four neighboring New England states were among the eight highest: New Hampshire (third highest, $5,953), Massachusetts (fifth highest, $5,381), Maine (sixth highest, $5,099), and Connecticut (eighth highest, $4,800).

**TABLE 1:** Case counts and costs of opioid use disorder and fatal opioid overdose and per capita cost, by jurisdiction — 38 states and the District of Columbia, 2017*

Jurisdiction^†^	Estimated case count of opioid use disorder	Case count of fatal opioid overdose	Cost of opioid use disorder, $ (millions)	Cost of fatal opioid overdose, $ (millions)	Combined cost of opioid use disorder and fatal opioid overdose, $ (millions)	Per capita cost of opioid use disorder, $	Per capita cost of fatal opioid overdose, $	Per capita combined cost of opioid use disorder and fatal opioid overdose, $
Hawaii	5,000	53	1,106.1	612.1	1,718.1	775	429	1,204
Minnesota	16,000	422	3,539.5	4,873.3	8,412.8	635	874	1,509
California	165,000	2,199	36,501.1	25,394.3	61,895.5	923	642	1,566
Wyoming	2,000	47	442.4	542.8	985.2	764	937	1,701
Texas	146,000	1,458	32,298.0	16,837.2	49,135.1	1,141	595	1,736
Iowa	17,000	206	3,760.7	2,378.9	6,139.6	1,196	756	1,952
Georgia	41,000	1,014	9,070.0	11,709.8	20,779.8	870	1,123	1,992
Mississippi	20,000	185	4,424.4	2,136.4	6,560.8	1,483	716	2,199
Colorado	35,000	578	7,742.7	6,674.8	14,417.5	1,381	1,190	2,571
Oklahoma	26,000	388	5,751.7	4,480.7	10,232.4	1,463	1,140	2,603
Oregon	37,000	344	8,185.1	3,972.6	12,157.7	1,976	959	2,935
New York	103,000	3,224	22,785.5	37,231.2	60,016.7	1,148	1,876	3,024
Missouri	34,000	952	7,521.4	10,993.8	18,515.3	1,230	1,798	3,029
Arizona	50,000	928	11,060.9	10,716.7	21,777.6	1,576	1,527	3,104
New Mexico	12,000	332	2,654.6	3,834.0	6,488.6	1,271	1,836	3,107
Washington	68,000	742	15,042.9	8,568.7	23,611.6	2,031	1,157	3,188
Wisconsin	36,000	926	7,963.9	10,693.6	18,657.4	1,374	1,845	3,219
Illinois	73,000	2,202	16,149.0	25,429.0	41,578.0	1,261	1,986	3,248
Florida	140,000	3,245	30,970.6	37,473.7	68,444.3	1,476	1,786	3,262
Virginia	63,000	1,241	13,936.8	14,331.2	28,268.0	1,645	1,692	3,337
South Carolina	37,000	749	8,185.1	8,649.5	16,834.6	1,629	1,722	3,351
Alaska	6,000	102	1,327.3	1,177.9	2,505.2	1,794	1,592	3,386
Tennessee	44,000	1,269	9,733.6	14,654.6	24,388.2	1,449	2,182	3,631
North Carolina	76,000	1,953	16,812.6	22,553.5	39,366.1	1,637	2,195	3,832
Utah	30,000	456	6,636.6	5,265.9	11,902.5	2,140	1,698	3,837
Vermont	5,000	114	1,106.1	1,316.5	2,422.6	1,774	2,111	3,884
Indiana	56,000	1,176	12,388.3	13,580.6	25,968.9	1,858	2,037	3,895
Nevada	34,000	412	7,521.4	4,757.8	12,279.3	2,509	1,587	4,096
Michigan	81,000	2,033	17,918.7	23,477.3	41,396.1	1,799	2,357	4,155
Rhode Island	6,000	277	1,327.3	3,198.8	4,526.1	1,253	3,019	4,271
District of Columbia	2,000	244	442.4	2,817.7	3,260.2	638	4,060	4,698
Connecticut	28,000	955	6,194.1	11,028.5	17,222.6	1,726	3,074	4,800
Maryland	30,000	1,985	6,636.6	22,923.0	29,559.6	1,097	3,788	4,884
Maine	12,000	360	2,654.6	4,157.3	6,812.0	1,987	3,112	5,099
Massachusetts	67,000	1,913	14,821.7	22,091.6	36,913.2	2,161	3,220	5,381
Kentucky	50,000	1,160	11,060.9	13,395.8	24,456.8	2,483	3,007	5,491
New Hampshire	14,000	424	3,097.1	4,896.4	7,993.5	2,306	3,646	5,953
Ohio	104,000	4,293	23,006.8	49,576.1	72,582.9	1,973	4,252	6,226
West Virginia	16,000	833	3,539.5	9,619.6	13,159.1	1,949	5,298	7,247

**Source:** Florence C, Luo F, Rice K. The economic burden of opioid use disorder and fatal opioid overdose in the United States, 2017. Drug Alcohol Depend 2021;218:108350. https://linkinghub.elsevier.com/retrieve/pii/S0376871620305159

* Estimated case counts of opioid use disorder in 2017 were extracted from the National Survey on Drug Use and Health’s 2-Year Restricted-Use Data Analysis System (2016–2017); cases of opioid use disorder were identified by using questions on opioid abuse or dependence during the past year; case counts of fatal opioid overdose and population estimates in 2017 were extracted from CDC’s WONDER database; cases of fatal opioid overdose were identified using *International Classification of Diseases, Tenth Revision* codes for the underlying cause-of-death (X40–X44, X60–X64, X85, and Y10–Y14) and then for multiple causes-of-death (T40.0–T40.4 and T40.6).

^†^ Jurisdictions are listed in ascending order of per capita combined cost of opioid use disorder and fatal opioid overdose.

**FIGURE:**
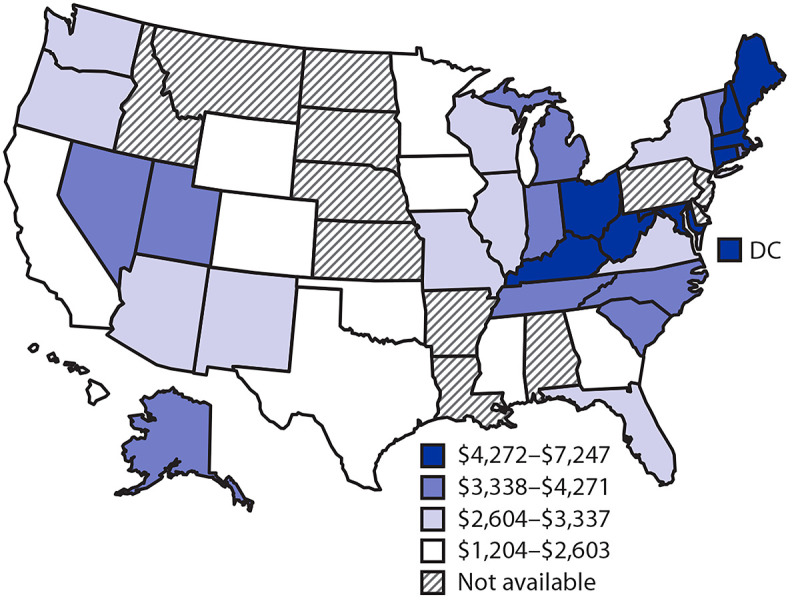
Per capita combined costs* of opioid use disorder and fatal opioid overdose — United States, 2017 **Abbreviation:** DC= District of Columbia. * Per capita combined costs are combined costs of opioid use disorder and fatal opioid overdose divided by state population and are expressed in 2017 U.S. dollars.

States with lower per capita combined costs were mainly in western regions: California, Hawaii, and Wyoming in the West; Minnesota in the Midwest; and Texas in the Southwest. Combined per capita costs were lowest in Hawaii ($1,204) and Minnesota ($1,509). Per capita cost of fatal opioid overdose was the lowest in Hawaii ($429), and per capita cost of opioid use disorder was the lowest in Minnesota ($635). The two most populous states (California and Texas) and the least populous state (Wyoming) were among the states with the lowest per capita combined costs: California, third lowest ($1,566), Wyoming, fourth lowest ($1,701) and Texas, fifth lowest ($1,736).

Reduced quality of life was the largest component of the cost of opioid use disorder, and the value of statistical life lost was the largest component of the cost of fatal opioid overdose (Table 2). These two components together accounted for approximately 84% of combined costs, followed by lost productivity.

**TABLE 2:** Cost components of opioid use disorder and fatal opioid overdose, by jurisdiction — 38 states and the District of Columbia, 2017*

Jurisdiction^†^	Estimated case counts of opioid use disorder	Cost components of opioid use disorder, $ (millions)					Case counts of fatal opioid overdose	Cost components of fatal opioid overdose, $ (millions)		
		Health care	Substance use treatment	Criminal justice	Lost productivity	Reduced quality of life		Health care	Lost productivity	Value of statistical life lost
Hawaii	5,000	73.5	8.3	34.8	73.5	915.9	53	0.3	76.5	535.3
Minnesota	16,000	235.3	26.6	111.4	235.3	2,931.0	422	2.3	609.0	4,262.0
California	165,000	2,426.4	273.9	1,148.5	2,426.6	30,225.7	2,199	12.0	3,173.5	22,208.8
Wyoming	2,000	29.4	3.3	13.9	29.4	366.4	47	0.3	67.8	474.7
Texas	146,000	2,147.0	242.4	1,016.2	2,147.2	26,745.2	1,458	8.0	2,104.1	14,725.1
Iowa	17,000	250.0	28.2	118.3	250.0	3,114.2	206	1.1	297.3	2,080.5
Georgia	41,000	602.9	68.1	285.4	603.0	7,510.6	1,014	5.5	1,463.4	10,240.9
Mississippi	20,000	294.1	33.2	139.2	294.1	3,663.7	185	1.0	267.0	1,868.4
Colorado	35,000	514.7	58.1	243.6	514.7	6,411.5	578	3.2	834.1	5,837.5
Oklahoma	26,000	382.3	43.2	181.0	382.4	4,762.8	388	2.1	559.9	3,918.6
Oregon	37,000	544.1	61.4	257.5	544.2	6,777.9	344	1.9	496.4	3,474.2
New York	103,000	1,514.7	171.0	716.9	1,514.8	18,868.2	3,224	17.6	4,652.7	32,560.8
Missouri	34,000	500.0	56.4	236.7	500.0	6,228.3	952	5.2	1,373.9	9,614.7
Arizona	50,000	735.3	83.0	348.0	735.3	9,159.3	928	5.1	1,339.2	9,372.4
New Mexico	12,000	176.5	19.9	83.5	176.5	2,198.2	332	1.8	479.1	3,353.0
Washington	68,000	1,000.0	112.9	473.3	1,000.1	12,456.6	742	4.1	1,070.8	7,493.8
Wisconsin	36,000	529.4	59.8	250.6	529.4	6,594.7	926	5.1	1,336.4	9,352.2
Illinois	73,000	1,073.5	121.2	508.1	1,073.6	13,372.6	2,202	12.0	3,177.8	22,239.1
Florida	140,000	2,058.8	232.4	974.5	2,059.0	25,646.0	3,245	17.7	4,683.0	32,772.9
Virginia	63,000	926.4	104.6	438.5	926.5	11,540.7	1,241	6.8	1,791.0	12,533.5
South Carolina	37,000	544.1	61.4	257.5	544.2	6,777.9	749	4.1	1,080.9	7,564.5
Alaska	6,000	88.2	10.0	41.8	88.2	1,099.1	102	0.6	147.2	1,030.2
Tennessee	44,000	647.0	73.0	306.3	647.1	8,060.2	1,269	6.9	1,831.4	12,816.3
North Carolina	76,000	1,117.6	126.2	529.0	1,117.7	13,922.1	1,953	10.7	2,818.5	19,724.4
Utah	30,000	441.2	49.8	208.8	441.2	5,495.6	456	2.5	658.1	4,605.4
Vermont	5,000	73.5	8.3	34.8	73.5	915.9	114	0.6	164.5	1,151.3
Indiana	56,000	823.5	93.0	389.8	823.6	10,258.4	1,176	6.4	1,697.1	11,877.0
Nevada	34,000	500.0	56.4	236.7	500.0	6,228.3	412	2.3	594.6	4,161.0
Michigan	81,000	1,191.1	134.5	563.8	1,191.3	14,838.1	2,033	11.1	2,933.9	20,532.3
Rhode Island	6,000	88.2	10.0	41.8	88.2	1,099.1	277	1.5	399.8	2,797.6
District of Columbia	2,000	29.4	3.3	13.9	29.4	366.4	244	1.3	352.1	2,464.3
Connecticut	28,000	411.8	46.5	194.9	411.8	5,129.2	955	5.2	1,378.2	9,645.0
Maryland	30,000	441.2	49.8	208.8	441.2	5,495.6	1,985	10.8	2,864.7	20,047.5
Maine	12,000	176.5	19.9	83.5	176.5	2,198.2	360	2.0	519.5	3,635.8
Massachusetts	67,000	985.3	111.2	466.4	985.4	12,273.5	1,913	10.4	2,760.7	19,320.4
Kentucky	50,000	735.3	83.0	348.0	735.3	9,159.3	1,160	6.3	1,674.1	11,715.4
New Hampshire	14,000	205.9	23.2	97.4	205.9	2,564.6	424	2.3	611.9	4,282.2
Ohio	104,000	1,529.4	172.6	723.9	1,529.5	19,051.3	4,293	23.4	6,195.4	43,357.2
West Virginia	16,000	235.3	26.6	111.4	235.3	2,931.0	833	4.6	1,202.1	8,412.9

**Source:** Florence C, Luo F, Rice K. The economic burden of opioid use disorder and fatal opioid overdose in the United States, 2017. Drug Alcohol Depend 2021;218:108350. https://linkinghub.elsevier.com/retrieve/pii/S0376871620305159

* Estimated case counts of opioid use disorder in 2017 were extracted from the National Survey on Drug Use and Health’s 2-Year Restricted-Use Data Analysis System (2016–2017); cases of opioid use disorder were identified by using questions on opioid abuse or dependence during the past year; case counts of fatal opioid overdose and population estimates in 2017 were extracted from CDC’s WONDER database; cases of fatal opioid overdose were identified by using *International Classification of Diseases, Tenth Revision* codes for the underlying cause-of-death (X40–X44, X60–X64, X85, and Y10–Y14) and then for multiple causes-of-death (T40.0–T40.4 and T40.6).

^†^ Jurisdictions are listed in ascending order of per capita combined cost of opioid use disorder and fatal opioid overdose.

## Discussion

The opioid overdose epidemic had a substantial economic impact on the United States during 2017. Individual states differed widely in overall and per capita economic cost. Per capita combined costs of opioid use disorder and fatal opioid overdose were highest in states in the Ohio Valley and New England regions. Three states in New England (Connecticut, Maine, and Massachusetts) had high per capita combined costs in 2017. However, previous reports have shown that these states had low per capita lifetime medical and work-loss costs from all fatal injuries (including opioid overdose) in 2014 (*7*). Further investigation is needed to ascertain why states that have relatively low costs for other types of injuries have relatively high costs related to opioid use disorder and fatal overdose.

Several effective strategies have been identified to improve opioid prescribing consistent with clinical guidelines, treat opioid use disorder, and prevent fatal overdose. Pain clinic laws and combined implementation of mandated provider review of state-run prescription drug monitoring program data have reduced the amounts of opioids prescribed and prescription opioid overdose death rates (*8*). Treatment with Food and Drug Administration–approved medications (methadone, buprenorphine, or naltrexone) is the most effective form of treatment for opioid use disorder (*9*). Overdose education and nasal naloxone distribution programs reduced opioid overdose mortality rates in Massachusetts (*10*).

The findings in this report are subject to at least four limitations. First, this study is limited to the 38 states and DC that met drug specificity requirements for mortality data, so the rankings of combined costs and per capita costs do not apply to all 50 states. Second, the cost of opioid use disorder was measured for a single year rather than a lifetime, even though opioid use disorder might have a long-lasting effect on a person’s life. Third, the estimated case counts of opioid use disorder likely underrepresent the true prevalence of opioid use disorder because NSDUH does not include persons who are incarcerated or experiencing homelessness, two groups that often have high rates of opioid use disorder. Finally, this study did not directly calculate state costs per case of opioid use disorder and fatal opioid overdose but rather applied the national costs per case of opioid use disorder and fatal opioid overdose to individual states. Population characteristics of opioid use disorder and fatal opioid overdose cases at the state level might differ from those at the national level, potentially biasing state cost estimates.

These estimated costs of opioid use disorder and fatal opioid overdose and their per capita costs at the state level can assist federal and state decision makers in understanding the magnitude of opioid use disorder and fatal opioid overdose in their jurisdictions. Federal and state public health agencies can use these data to help guide decisions regarding research, prevention and response activities, and resource allocation.

**Summary**

**What is already known about this topic?**

The U.S. economic cost of opioid use disorder ($471 billion) and fatal opioid overdose ($550 billion) during 2017 totaled $1,021 billion.

**What is added by this report?**

In the 39 jurisdictions studied, combined costs of opioid use disorder and fatal opioid overdose varied from $985 million in Wyoming to $72,583 million in Ohio. Per capita combined costs varied from $1,204 in Hawaii to $7,247 in West Virginia. States with high per capita combined costs were located mainly in the Ohio Valley and New England.

**What are the implications for public health practice?**

Federal and state public health agencies can use these data to help guide decisions regarding research, prevention and response activities, and resource allocation.

## Footnotes

* NSDUH classified respondents as having opioid use disorder during the past year if they had a heroin use disorder (dependence or abuse), pain reliever use disorder (dependence or abuse), or both during the past year.

† Cases of fatal opioid overdose include all opioid-related overdose deaths regardless of intent (intentional, unintentional, homicide, or undetermined).

§ Cost estimates per case for components of opioid use disorder based on the value of a statistical life were as follows: health care, $14,705; substance use treatment, $1,660; criminal justice, $6,961; lost productivity, $14,707; and reduced quality of life, $183,186. Cost estimates per case for components of fatal opioid overdose were as follows: health care, $5,462; lost productivity, $1.443 million; and value of statistical life, $10.1 million.
